# Voiceprint Fault Diagnosis of Converter Transformer under Load Influence Based on Multi-Strategy Improved Mel-Frequency Spectrum Coefficient and Temporal Convolutional Network

**DOI:** 10.3390/s24030757

**Published:** 2024-01-24

**Authors:** Hui Li, Qi Yao, Xin Li

**Affiliations:** School of Electrical Engineering, Xi’an University of Technology, Xi’an 710048, China; 2211921149@stu.xaut.edu.cn (Q.Y.); 2211921145@stu.xaut.edu.cn (X.L.)

**Keywords:** converter transformer, current, fault diagnosis, improved hunter–prey optimization, multi-strategy improved MFCC, voiceprint signal

## Abstract

In order to address the challenges of low recognition accuracy and the difficulty in effective diagnosis in traditional converter transformer voiceprint fault diagnosis, a novel method is proposed in this article. This approach takes account of the impact of load factors, utilizes a multi-strategy improved Mel-Frequency Spectrum Coefficient (MFCC) for voiceprint signal feature extraction, and combines it with a temporal convolutional network for fault diagnosis. Firstly, it improves the hunter–prey optimizer (HPO) as a parameter optimization algorithm and adopts IHPO combined with variational mode decomposition (VMD) to achieve denoising of voiceprint signals. Secondly, the preprocessed voiceprint signal is combined with Mel filters through the Stockwell transform. To adapt to the stationary characteristics of the voiceprint signal, the processed features undergo further mid-temporal processing, ultimately resulting in the implementation of a multi-strategy improved MFCC for voiceprint signal feature extraction. Simultaneously, load signal segmentation is introduced for the diagnostic intervals, forming a joint feature vector. Finally, by using the Mish activation function to improve the temporal convolutional network, the IHPO-ITCN is proposed to adaptively optimize the size of convolutional kernels and the number of hidden layers and construct a transformer fault diagnosis model. By constructing multiple sets of comparison tests through specific examples and comparing them with the traditional voiceprint diagnostic model, our results show that the model proposed in this paper has a fault recognition accuracy as high as 99%. The recognition accuracy was significantly improved and the training speed also shows superior performance, which can be effectively used in the field of multiple fault diagnosis of converter transformers.

## 1. Introduction

In order to ensure the secure and reliable operation of converter transformers, which serve as intermediate devices for AC-DC power transmission technology, it is essential to conduct research on fault diagnosis. This research aims to enhance the accuracy and speed of fault identification, helping to promptly detect internal defects and prevent the further escalation of accidents. Unlike regular power transformers, converter transformers operate in a unique AC-DC working environment, which implies a higher level of harmonic currents. Consequently, this complexity in operational characteristics presents challenges in employing conventional fault diagnosis methods designed for traditional power transformers [1,2].

The converter transformer, in the process of operation with the core and windings, produces vibration because of electric power and other factors, and thus mechanical wave propagation through the transformer oil and rigid connection to the box. The resulting vibration and voiceprint signals contain a large amount of state information based on the vibration signal monitoring means that are widely used in the online monitoring of power equipment [3,4]. In the vibration signal acquisition process, the deployment location requirements of sensors are strict. Smaller deviations will interfere with the results. The noise detection method is used as a non-contact measurement; its sensor installation is convenient for solving the problem of high spatial sensitivity. At the same time, the voiceprint signal acquisition device has a wide frequency range to meet the monitoring requirements of different specifications of the transformer [5,6]. Numerous scholars both domestically and internationally have conducted research in this area, achieving promising results. In reference [7], four voiceprint emission feature spectra were constructed, and a lightweight fault diagnosis model was established to diagnose loose winding faults in transformers. Reference [8], based on the no-load operation of transformers, employed MFCC for voiceprint feature extraction, introduced Principal Components Analysis (PCA) to remove redundant features, and ultimately utilized the Vector Quantization (VQ) algorithm for accurate identification of loosened iron core faults. Reference [9] extracted features of on-load tap changers using Mel spectrograms and combined them with convolutional neural networks to recognize mechanical faults. However, the abovementioned voiceprint emission recognition techniques are based on traditional MFCC, which involves a cumbersome process of frame segmentation, windowing, and Fourier transformation to overcome spectral leakage issues. Furthermore, due to the inherent limitations of single-channel signal sources, the practicality of fault diagnosis using voiceprint emission signals is mostly limited to single-fault diagnosis.

To address the issue of the single-fault feature, reference [10] utilized Complete Ensemble Empirical Mode Decomposition (CEEMD) and short-time Fourier transform (STFT) to obtain temporal and spectral information about the signals. Deep fault features were then extracted using a deep fused convolutional neural network (DFCNN). Similarly, reference [11] proposed a mixed algorithm called high-order singular value decomposition (HOSVD)–high-order alternation least square (HOALS) to extract multi-dimensional features for pattern recognition. Furthermore, reference [12] combined the fusion multiscale convolutional neural network (F-MSCNN) to fuse sound and vibration features, leveraging the learning of multi-scale features for subsequent classification. Reference [13] proposed a real-time fault diagnostic method for hydraulic systems using data collected from multiple sensors in order to overcome the lack of information contained in a single sensor. Reference [14] processed signals from multiple sensors, thereby expanding the number of samples to enhance the diagnostic performance. However, most of the existing studies are based on single or homogeneous signals. They focus on extracting multidimensional features from different angles without considering multiple signal sources. The above diagnostic models do not start from different types of signal sources and ignore the correlation between different signals, making it difficult to extract deep information effectively from faults.

Existing approaches on data-driven fault classification mostly rely on artificial intelligence algorithms to analyze historical data and extract fault features, and the selection of parameters during the model training process has a crucial impact on the accuracy and convergence speed of fault classifiers. Reference [15] proposed a novel expectation maximization-unscented particle filter-Wilcoxon rank sum test (EM-UPF-W) method for data-driven techniques, which adaptively estimates noise variables with the help of the EM algorithm. References [16,17] used an artificial intelligence optimization algorithm for the adaptive optimization of machine learning parameters to avoid the human experience of parameter selection, but the existing artificial intelligence optimization is prone to the problem of local optimal stagnation, which has an impact on the final convergence speed and accuracy of the model.

Given this context, this article is focused on the division of current signals into intervals, combining voiceprint signals to achieve fault diagnosis in converter transformers. It overcomes the inherent limitations of single signal sources and conducts research on multi-fault diagnosis. The IHPO method is proposed to effectively address the local optimization problem, serving as a subsequent parameter optimization algorithm. VMD is employed for noise reduction, while the S-transform is utilized as a time-frequency conversion method. The improved MFCC technique based on multiple strategies is employed for feature extraction. ITCN is utilized for accurate fault identification, offering a novel approach for fault diagnosis in converter transformer systems. Furthermore, a specific 800 kV converter station was taken as a case study to validate the effectiveness of this integrated model. 

The main contributions of this article are summarized as follows:

- This paper aims to counteract the problems of the traditional hunter–prey optimization algorithm, which easily falls into the local optimum, and of which the traversal of population initialization is not strong. It is improved via the introduction of SPM chaotic mapping and the Levy flight strategy, which is used for the adaptive selection of parameters in the fault diagnostic model to avoid the interference of the human experience selection.
- Multi-strategy improved MFCC is proposed for extracting voiceprint signals from converter transformers. Compared with the traditional voiceprint signal feature extraction method, the proposed approach incorporates the characteristics specific to the voiceprint signals of electric power equipment. It overcomes the interference of redundant information and demonstrates enhanced feature extraction capabilities.
- This paper introduces load signals to segment the operational intervals of converter transformers, realizes fault diagnosis through multiple types of signal sources, and proposes the improved multi-strategy MFCC and IHPO-VMD-ITCN fault diagnostic models. The experimental results demonstrate that the proposed fault diagnostic methods exhibit significant improvements in terms of both accuracy and calculation speed.

## 2. Analysis of Vibration Mechanism of Converter Transformer

Similarly to traditional power transformers, the vibration of converter transformers is induced by the electromagnetic forces in the windings and the expansion and contraction of the core due to magnetic hysteresis. These vibrations propagate through the transformer oil and rigid connections to the enclosure. However, owing to the complex environment resulting from the dual impact of alternating and direct currents, the vibration excitations are often characterized by multiple harmonic frequencies, leading to intricate vibration patterns in different areas.

### 2.1. Winding Vibration Mechanism Analysis

In accordance with the principles of high-voltage transmission, the current in converter transformers is accompanied by harmonic currents, including the 6k+1(k=1,2,3,…) th harmonic current at 50 Hz. This is manifested in Equation (1).

(1) $$i=\sum I_{\alpha m}\cos(\alpha \omega_{1}t+\Phi_{\alpha })$$

where $I_{\alpha m}$ is the amplitude of each harmonic current, $\Phi_{\alpha }$ is the phase angle of each harmonic, and $\omega_{1}$ is the angular frequency of the 50 Hz current.

The interaction between currents of varying frequencies and magnetic fields generates axial and radial electromagnetic forces is expressed in Equation (2). The windings vibrate under the influence of these electromagnetic forces.

(2) $$\begin{array}{l} F_{a}=k_{fa}{\left(\sum I_{\alpha m}\cos(\alpha \omega_{1}t+\Phi_{\alpha })\right)}^{2}\\ F_{r}=k_{fr}{\left(\sum I_{\alpha m}\cos(\alpha \omega_{1}t+\Phi_{\alpha })\right)}^{2} \end{array}$$

where $k_{fa}$ and $k_{fr}$ represent the axial and radial electromagnetic force coefficient and $F_{a}$ and $F_{r}$ represent the winding axial and radial electromagnetic force.

Based on the motion differential equation, the acceleration of winding vibration can be represented by Equation (3):

(3) $$\begin{array}{l} a_{a}=k_{aa}(\sum p_{1}{I_{\alpha m}}^{2}\cos(2\alpha \omega_{1}t+\varphi_{1})+\sum_{2}p_{2}I_{\alpha_{1}m}\cos((\alpha_{1}+\alpha_{2})\omega_{1}t+\varphi_{2})\\ +\sum_{2}p_{3}I_{\alpha_{1}m}I_{\alpha_{2}m}\cos((\alpha_{1}-\alpha_{2})\omega_{1}t+\varphi_{3}))\\ a_{r}=k_{ar}(\sum p_{1}{I_{\alpha m}}^{2}\cos(2\alpha \omega_{1}t+\varphi_{1})+\sum_{2}p_{2}I_{\alpha_{1}m}\cos((\alpha_{1}+\alpha_{2})\omega_{1}t+\varphi_{2})\\ +\sum_{2}p_{3}I_{\alpha_{1}m}I_{\alpha_{2}m}\cos((\alpha_{1}-\alpha_{2})\omega_{1}t+\varphi_{3})) \end{array}$$

where $\sum_{2}$ is the sum of multiplication of different harmonics, $k_{aa}$ and $k_{ar}$ are the axial and radial acceleration coefficients, $p_{1}$ , $p_{2}$ , and $p_{3}$ are the calculation parameters, $\alpha_{1},\alpha_{2}$ are the number of harmonics, and $\varphi_{1}$ , $\varphi_{2}$ , and $\varphi_{3}$ are the acceleration phase angles.

From Equation (3), it can be observed that under the influence of the $6k_{1}+1(k_{1}=1,2,3,\ldots )$ th harmonic, apart from the 100 Hz component, there is also a significant presence of the $100k_{1}\mathrm{Hz}$ th harmonic in the vibration of the converter transformer. When the natural frequency of the windings is close, resonance can easily occur, leading to a deviation of the dominant vibration frequency from 100 Hz.

### 2.2. Core Vibration Mechanism Analysis

The vibration of the core is primarily induced by magnetostriction. Furthermore, the excitation voltage of the converter transformer contains numerous harmonic components. Taking the influence of harmonic voltages into account, the vibration of the core can be represented by Equation (4):

(4) $$\begin{array}{l} a=\frac{d^{2}(△L)}{dt^{2}}=k_{a}(\sum 2{U_{\alpha m}}^{2}\cos(2\alpha \omega_{1}t+\varphi_{\alpha })+\sum_{2}q_{1}\cos((\alpha_{1}+\alpha_{2})\omega_{1}t+(\varphi_{\alpha 1}+\varphi_{\alpha 2}))\\ +\sum_{2}q_{1}\cos((\alpha_{1}-\alpha_{2})\omega_{1}t+(\varphi_{\alpha 1}-\varphi_{\alpha 2})) \end{array}$$

Among them:

(5) $$\begin{array}{l} q_{1}=\frac{{\left(\alpha_{1}+\alpha_{2}\right)}^{2}U_{\alpha_{1m}}U_{\alpha_{2m}}}{\alpha_{1}\alpha_{2}}\\ q_{2}=\frac{{\left(\alpha_{1}-\alpha_{2}\right)}^{2}U_{\alpha_{1m}}U_{\alpha_{2m}}}{\alpha_{1}\alpha_{2}} \end{array}$$

where $U_{\alpha m}$ is the amplitude of each voltage harmonic, △L is the magnetostrictive deformation of the silicon steel sheet, and $k_{a}$ is the saturation flux coefficient.

From Equation (4), it can be observed that the dominant frequency of the core vibration is primarily at 100 Hz. The influence of harmonics introduce a significant presence of the $100k_{1}\;\mathrm{Hz}$ harmonic components. However, nonlinearities in the core and other factors may lead to deviations in vibration.

### 2.3. Fault Voiceprint Characterization of Converter Transformers

Similarly to ordinary power transformers, converter transformers are mainly composed of iron core, windings, and rigid connectors. When the iron core ages or experiences transportation and installation before operation, iron core loosening may occur. If the condition of iron core loosening is not promptly addressed, it will continue to accumulate, ultimately leading to iron core loosening failure. Iron core loosening failure results in a decrease in the fastening force between the silicon steel sheets of the iron core, thereby increasing the air gap between the stacked pieces. This causes a significant rise in the amplitude of iron core vibration acceleration, leading to changes in the intrinsic frequency of vibration and altering the voiceprint characteristics of the transformer. Similarly, during operation, the converter transformer is constantly subjected to the impact of electric power. In the event of a short-circuit fault, the intensification of electric power can prompt the occurrence of winding loosening faults. This leads to an aggravation of axial vibration, a significant increase in vibration acceleration amplitude, and changes in the vibration frequency distribution, resulting in alterations to the voiceprint characteristics of the transformer. When the converter transformer is running under bias magnetic conditions, the current signal can be regarded as the superposition of a DC component and Equation (1); according to Section 2.1 and Section 2.2 of the core and winding vibration mechanism analysis, it can be observed that, at this time, the vibration frequency of the converter transformer changes significantly.

In summary, when a fault occurs in the converter transformer, its core and winding vibration change significantly. The fault voiceprint signal generated under these conditions differs from that of normal operation. Therefore, the fault diagnosis of the converter transformer can be realized by adopting a machine learning algorithm for effective feature extraction of the voiceprint signal.

### 2.4. Characterization of Voiceprint Pattern Changes under Operating Conditions

The voiceprint signal and vibration signal, originating from the same source, exhibit a strong correlation. Based on the analysis in Section 2.1 and Section 2.2, this study delves into the vibration characteristics of converter transformers during operation.

This study focuses on 28 converter transformers in a specific 800 kV converter station. Among them, there are 12 transformers per pole and 4 transformers on standby. The parameters of certain converter transformers are presented in Table 1.

**Figure 1:**
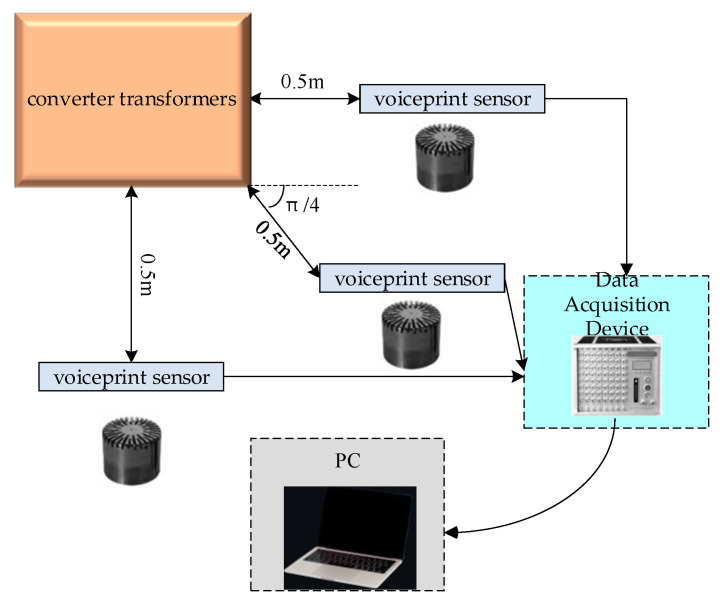
Voiceprint data acquisition system.

**Figure 2:**
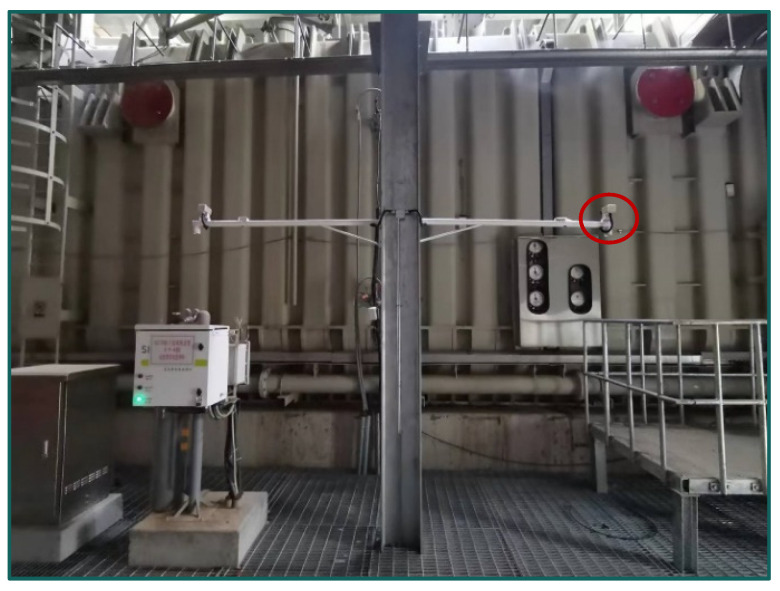
On-site acquisition.

The voiceprint signal acquisition system for the converter transformers is illustrated in Figure 1, and on-site acquisition photos are presented in Figure 2. We employed a combination of HS14401 capacitive sound sensors with a sampling frequency of 16 kHz along with a DHDAS dynamic signal acquisition instrument. Each converter transformer is equipped with three voiceprint acquisition devices, positioned on both sides and at a 45-degree angle, 0.5 m away from the enclosure. The data were collected in the outdoor substation environment under normal operating conditions, which may include noise interference. The voiceprint acquisition system was configured to collect voiceprint signals every 30 min, with each collection lasting for 60 s. Electrical parameters within the converter station were recorded every 30 min to ensure synchronization between the voiceprint signals and electrical parameters.

**Figure 3:**
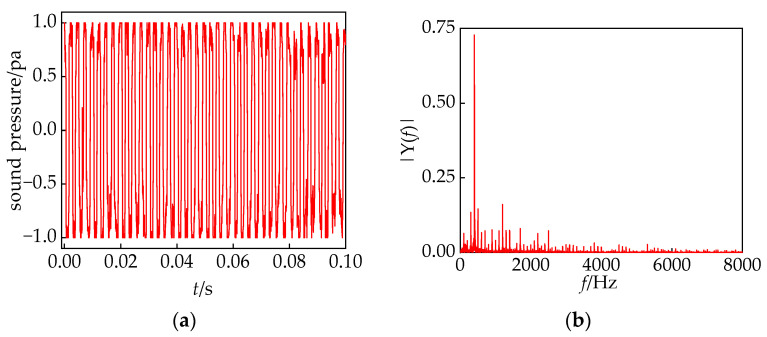
(**a**) Time domain characteristics; (**b**) frequency domain characteristics.

We selected time-length 0.1 s converter transformer in-operation voiceprint slices as the object of study. The time-domain and frequency-domain characteristics are illustrated in Figure 3. The main frequency of the converter transformer is 400 Hz, accompanied by a significant number of harmonics. This is attributed to the proximity of the winding intrinsic frequency to 400 Hz and the resonance of the converter transformer $100k_{1}\;\mathrm{Hz}$ component, resulting in a deviation of 100 Hz compared to ordinary power transformers. This deviation corresponds to the theoretical analysis mentioned above.

**Figure 4:**
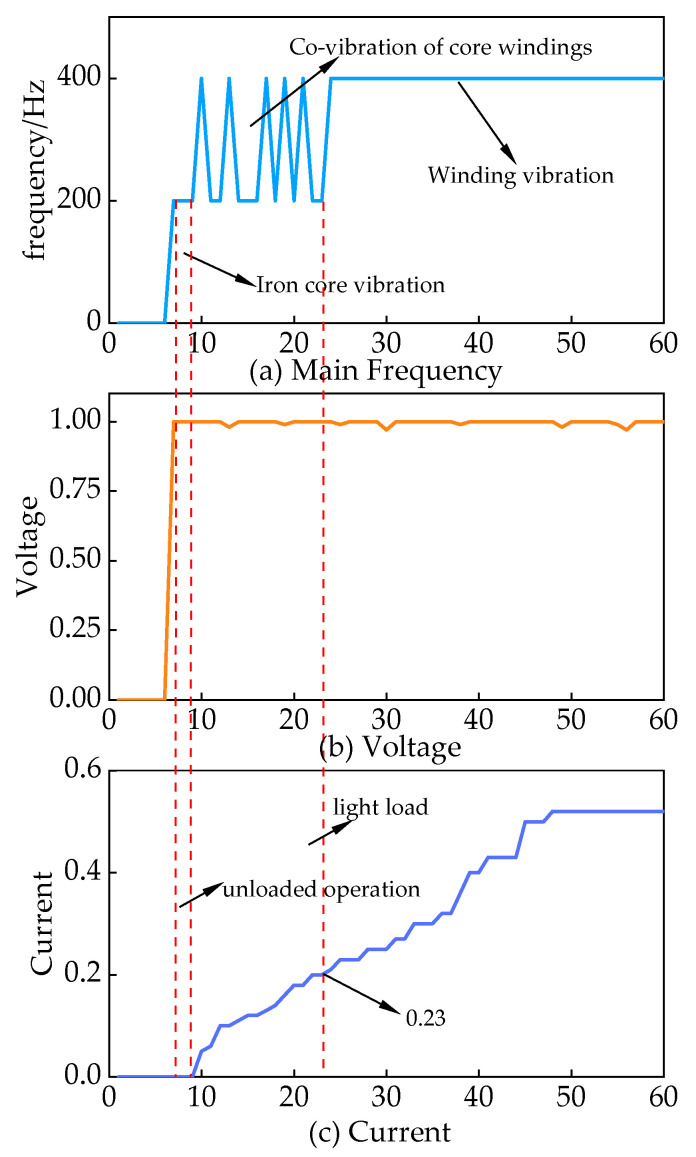
Characteristics of the main frequency of the voiceprint signal of the converter transformer with the variation in current.

The vibration characteristics of converter transformers vary under different operating conditions. In a no-load converter transformer, the core winding resonance becomes prominent. Under heavy load, the dominant vibration shifts to winding [18,19,20]. To facilitate a more precise quantitative analysis, this article focuses on the high-end Y/D converter transformer of pole II. The main objective is to analyze the main frequency change pattern of voiceprint characteristics concerning the magnitude of current. The results are depicted in Figure 4. Under no load, the main frequency of the converter transformer is 200 Hz, indicating the core vibration stage. At the rated voltage, when the valve side current is less than 0.2 $I_{N}$ , the main frequency alternates between 200 Hz and 400 Hz. During this period, the core winding dominance alternates. However, when the current exceeds 0.23 $I_{N}$ , the main frequency stabilizes at 400 Hz, signifying the dominance of winding vibration.

Based on the information provided, a strong correlation exists between the electrical signals and voiceprint features of converter transformers. The division of converter transformers into three interval states, as illustrated in Table 2, allows for a phased approach to fault diagnosis. This approach proved effective in overcoming the issue of overlapping between core faults and winding faults, ultimately enhancing the accuracy of fault identification.

## 3. Description of Fault Diagnosis Algorithms

### 3.1. Improved Hunter–Prey Optimization Algorithms

The hunter–prey optimization algorithm is a new intelligent optimization algorithm proposed by Naruei et al. in 2021 [21]. In this algorithm, the hunter adjusts its position to obtain the best hunting position, while the prey moves to a safe position to avoid the hunter’s attack, and the safest position of the prey is the optimal solution of the problem to be optimized. This article proposes an improvement of the HPO algorithm by introducing the Levy flight strategy and SPM chaotic mapping. The modifications are briefly described as follows.

(1) Initialization: The conventional HPO algorithm achieves population initialization using Equation (6), as described below:
(6) $$x_{i}=rand(1,d)\times (ub,lb)+lb$$
  wherein $x_{i}$ represents the positions of hunters or prey, *d* represents the problem dimensionality, and ub , lb represent the upper and lower bounds of the problem.

**Figure 5:**
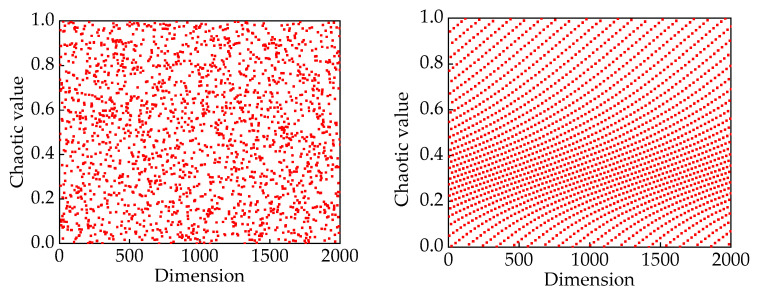

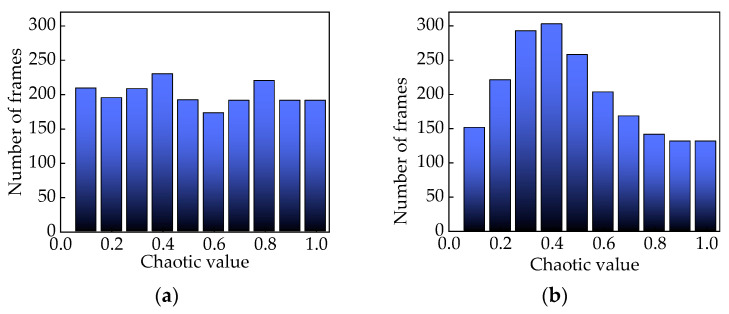
(**a**) SPM chaotic mapping value distribution; (**b**) circle chaotic mapping value distribution.

We chose Strongly Perturbed Mix (SPM) chaotic mapping for initializing the population, as shown in Figure 5. In comparison to circle mapping, the SPM demonstrates enhanced randomness and tergodicity, effectively addressing the issue of local clustering of individual hunters and prey [22]. The expression for SPM chaotic mapping is given by Equation (7).

(7) $$x_{i+1}=\left\{ \begin{array}{l} \mathrm{mod}(\frac{x(t)}{\eta })+\mu \sin(\pi x(t)+r,1),\\ 0\leq x(t)\leq \eta \\ \mathrm{mod}(\frac{x(t)/\eta }{0.5-\eta })+\mu \sin(\pi x(t)+r,1),\\ \eta \leq x(t)\leq 0.5\\ \mathrm{mod}(\frac{1-x(t)/\eta }{0.5-\eta })+\mu \sin(\pi (1-x(t))+r,1),\\ 0.5\leq x(t)\leq 1-\eta \\ \mathrm{mod}(\frac{1-x(t)}{0.5})+\mu \sin(\pi (1-x(t))+r,1),\\ 1-\eta \leq x(t)\leq 1 \end{array}\right.$$

In Equation (7), the parameter η⊆(0,1),μ⊆(0,1) is typically chosen within the range of (0.4, 0.3).

(2) Optimization strategy: Hunters select prey that are far away from the group as their search targets, while the prey continuously move to evade hunter attacks and maximize their chances of survival. The position update for hunters and prey can be described by Equations (8) and (9), respectively.
(8) $$\begin{array}{l} x_{i,j}(t+1)=x_{i,j}(t)+0.5[(2CZP_{pos(j)}-x_{i,j}(t))+\\ \left(2(1-C)Z\mu -x_{i,j}(t))\right] \end{array}$$
  wherein $x_{i,j}(t+1)$ represents the position of the *i*th hunter in the *j*th dimension at the (*t* + 1)th iteration, $x_{i,j}(t)$ represents the position of the *i*th hunter at the *t*th iteration, $P_{pos(j)}$ represents the position of the prey in the *j*th dimension, C=1−0.98t/T represents the balance parameter between exploration and exploitation, and *Z* is an adaptive parameter.
(9) $$x_{i,j}(t+1)=T_{pos(j)}+CZ\cos(2\pi R_{1})\cdot (T_{pos(j)}-x_{i,j}(t))$$
  wherein $T_{pos(j)}$ represents the global best position and $R_{1}$ represents a random number within the range of [−1, 1].

It is challenging to overcome local optima solely by introducing SPM chaotic mapping. However, the utilization of the Levy flight strategy allows for a quick escape from local optima. The implementation approach is depicted in Equation (10).

(10) $$Levy(s)\approx \frac{\lambda \beta (\Gamma (\lambda ))\sin(\frac{\pi \lambda }{2})}{\pi }\cdot \frac{1}{s^{1+\lambda }}$$

wherein $\Gamma (\lambda )=\int_{0}^{\infty }t^{z-1}e^{-t}dt$ and the value of β is set to 1.5.

In practical applications, the Mantegna method is commonly used to generate random step lengths following a Levy distribution, as described in Equations (11) and (12).

(11) $$S=\frac{\mu }{{\left|v\right|}^{\frac{1}{\beta }}}$$

(12) $$\begin{array}{l} \mu ∼N(0,\sigma^{2}),v∼N(0,1)\\ \sigma ={\left\{\frac{\Gamma (1+\beta )\sin(\frac{\pi \beta }{2})}{\beta \Gamma (\frac{1+\beta }{2})2^{\frac{\beta -1}{2}}}\right\}}^{\frac{1}{\beta }} \end{array}$$

In the IHPO optimization algorithm, if the change in fitness values is continuously less than 0.001, the Levy flight strategy aids in escaping local optima. This generates the candidate solution for the next iteration, as shown in Equation (13).

(13) $$x_{i}^{t+1}=x_{i}^{t}+\theta ⊕Levy(\beta )$$

In the equation, ⊕ denotes element-wise multiplication, θ is a random number uniformly distributed in the range [0, 1], and β is equal to 1.5.

The pseudocode used to improve the hunter–prey optimization algorithm is as follows in Algorithm 1:
**Algorithm 1** Improve hunter–prey optimization
**Input:** HPO Parameters
**Output:** TargetScore, Best pos, Convergence curve
1: Initialize Hppos
2: Evaluate fitness of each HPpos 
3: Set Target as the best HPpos, TargetScore as its fitness 
4: **for** *t* = 2 **to** Max_iteration **do**
5:    Update *c*

6:    Update kbest 
7:    **for** *i* = 1 **to** *N* **do**

8:       Generate random numbers 
9:       **if** rand < B **then**

10:        Calculate xi and dist 
11:        Set SI as HPpos(idxsortdist(kbest)) 
12:        Update HPpos(*i*,:) using formula with levy, l, c, z, SI, xi 
13:      **else**

14:        **for**
*j* = 1 to dim do 
15:           Calculate v and rr 
16:           Update HPpos(*i*,*j*) using formula with z(*j*), rr, Target(*j*), HPpos(*i*,*j*) 
17:        **end for**

18:      **end if**

19:      Clip HPpos(*i*,:) values to be within bounds of lb and ub 
20:      Evaluate fitness of HPpos(*i*,:) 
21:      **if** HPposFitness(*i*) < TargetScore **then**

22:        Update Target and TargetScore 
23:      **end if**

24:    **end for**

25:    Store TargetScore in Convergence curve(*t*) 
26: **end for**

**Figure 6:**
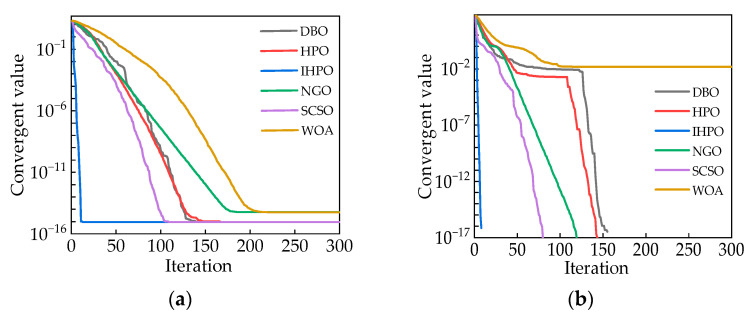
(**a**) Comparison of the optimization performance of the measurement function (14); (**b**) comparison of the optimization performance of the measurement function (15).

To validate the superiority of the IHPO algorithm, this article compares its performance with traditional optimization algorithms using the test function described in Equations (14) and (15). The results are depicted in Figure 6.

(14) $$\begin{array}{l} f_{1}(x)=-20\exp(-0.2\sqrt{\frac{1}{n}\sum_{i=1}^{n}x_{i}^{2}})-\\ \exp(\frac{1}{n}\sum_{i=1}^{n}\cos(2\pi x_{i}))+20+e \end{array}$$

(15) $$f_{2}(x)=\frac{1}{4000}\sum_{i=1}^{n}(x_{i}^{2})-\prod_{i=1}^{n}\cos(\frac{x_{i}}{\sqrt{i}})+1$$

According to Figure 6a,b, it can be observed that the IHPO optimization algorithm converges to values of $8.9\times 10^{-16}$ and 0, respectively. The convergence speed of the IHPO algorithm is significantly higher than that of other traditional algorithms, achieving superior convergence values with the fewest number of iterations.

### 3.2. Variational Mode Decomposition

During the process of collecting transformed voiceprint signals, there is often a significant amount of noise interference. In order to ensure the accuracy of fault diagnosis, this article adopts the VMD algorithm for denoising processing, aiming to restore the original voiceprint signal as faithfully as possible.

The VMD algorithm constructs a variational problem and solves it [23,24]. Firstly, the original signal is decomposed into *k* modal components, denoted as $\mu_{k}(t)$ . The energy spectrum is obtained through Hilbert transformation. f(t) is made equal to each modal component $\mu_{k}(t)$ as a constraint condition, and the Lagrange multiplier λ(t) and penalty factor α are introduced to transform it into a variational problem, as shown in Equation (16).

(16) $$\left\{ \begin{array}{l} \underset{\left\{\mu_{k}\}\{\omega_{k}\right\}}{\min}\left\{{\sum_{k}^{K}\left\|\partial_{t}[(\delta (t)+\frac{j}{\pi t})*\mu_{k}(t)]e^{-j\omega_{k}t}\right\|}_{2}^{2}\right\}\\ \underset{\mu_{k},\omega_{k}\lambda }{L}=\alpha {\sum_{k}^{K}\left\|\partial_{t}[(\delta (t)+\frac{j}{\pi t})*\mu_{k}(t)]e^{-j\omega_{k}t}\right\|}_{2}^{2}\\ +{\left\|f(t)-\sum_{k=1}^{K}\mu_{k}(t)\right\|}_{2}^{2}+\langle \lambda (t),f(t)-\sum_{k=1}^{K}\mu_{k}(t)\rangle \end{array}\right.$$

In Equation (16), * represents the convolution operation, $\mu_{k}(t)$ is the *k*-th modal component, $\omega_{t}$ is the central frequency, δ(t) is the impulse function, $\partial_{t}$ represents the partial derivative with respect to t, and $\langle \lambda (t),f(t)-\sum_{k=1}^{K}\mu_{k}(t)\rangle$ denotes the inner product.

The alternating direction multiplier method is used to solve the variational problem to find the optimal values of $\mu_{k}(t)$ , $\omega_{k}$ , which is realized in the following steps.

(1) Initialize the parameters $\mu_{k}(t)$ , $\omega_{k}$ , λ , set the loop n=n+1 , and iteratively update the parameters according to Equations (17)–(19).
(2) Update $\mu_{k}(t)$ .
(17) $${\hat{\mu }}_{k}^{n+1}(\omega )=\frac{\hat{f}(\omega )-\sum_{i=1}^{k-1}{\hat{\mu }}^{n}(\omega )+\frac{{\hat{\lambda }}^{n}(\omega )}{2}}{1+2\alpha {\left(\omega -\omega_{k}^{n}\right)}^{2}}$$
  In Equation (17), ${\hat{\mu }}_{k}^{n+1}(\omega )$ , $\hat{f}(\omega )$ , ${\hat{\lambda }}^{n}(\omega )$ are the Fourier transforms corresponding to $\mu_{k}^{n+1}$ , f(t) , $\lambda^{n}$ .
(3) Update $\omega_{k}$ .
(18) $$\omega_{k}^{n+1}=\frac{\int_{0}^{\infty }\omega {\left|{\hat{\mu }}_{k}^{n+1}(\omega )\right|}^{2}d\omega }{\int_{0}^{\infty }{\left|{\hat{\mu }}_{k}^{n+1}(\omega )\right|}^{2}d\omega }$$
(4) Update λ .
(19) $${\hat{\lambda }}^{n+1}={\hat{\lambda }}^{n}+\tau [\hat{f}(\omega )-\sum_{k=1}^{K}{\hat{\mu }}_{k}^{n+1}(\omega )]$$
(5) Determine convergence.
(20) $$\frac{\sum_{k=1}^{K}{\left\|{\hat{\mu }}_{k}^{n+1}-{\hat{\mu }}_{k}^{n}\right\|}_{2}^{2}}{{\left\|{\hat{\mu }}_{k}^{n+1}\right\|}_{2}^{2}}<\varsigma$$
  by setting ς>0 .
(6) Determine whether the iteration condition is satisfied; if not, return to step (2).

### 3.3. Multi-Strategy Improvement of MFCC for Dimensionality Reduction Extraction of Voiceprint Features

As a common speech feature extraction method, MFCC is widely used in the field of speech recognition [25]. Considering that spectral leakage in the Fourier transform is very likely to occur, the S-transform is used as a time-frequency conversion method, and combined with the characteristics of the stationary energy of the converter voiceprint signal, it undergoes processing in the medium time to obtain the improved MFCC method to realize the voiceprint signal feature extraction.

#### 3.3.1. S-Transform

The S-transform employs the Gaussian window function with adaptive adjustment of time and frequency parameters, replacing the fixed window function of the Fourier transform and the scale parameter window function of the wavelet transform. This approach exhibits higher-frequency characteristics at low frequencies and effectively improves the shortcomings of the Fourier transform [26].

The result of signal x(t) after S-transformation is shown in Equation (21).

(21) $$S(\tau ,f)=\int_{-\infty }^{+\infty }x(\eta )w(\eta -\tau ,f)e^{-j2\pi f\eta }d\eta$$

where *f* is the frequency, η is the time variable of x(η) , τ is the time component after S-transformation, and w(η−τ,f) is the Gaussian window function for adaptive adjustment, as shown in Equation (22):

(22) $$w(\eta -\tau ,f)=\frac{\left|f\right|}{\sqrt{2\pi }}e^{-\frac{{\left(t-\eta \right)}^{2}f^{2}}{2}}$$

#### 3.3.2. Multi-Strategy Improvement MFCC

In the field of audible sound recognition, given that the human ear exhibits varying sensitivities to the perception of each frequency band and the perception of the normal frequency band is nonlinear, Mel filtering is typically employed to transform the spectral information of voiceprint into Mel spectrum under Mel scale. The relationship between the normal frequency scale and the Mel frequency scale is expressed as in Equation (23):

(23) Mel(k)=2595×lg(1+f/700)

where *f* is the frequency on the regular scale and *k* is the frequency scale on the Mel scale.

In the domain of power equipment fault diagnosis, low-frequency information within 1000 Hz frequently incorporates numerous fault characteristics. Consequently, the utilization of Mel filters can adjust voiceprint information to varying degrees, enhance low-frequency information, and filter high-frequency information and compress it. The equal-height Mel filter bank function is expressed in Equation (24):

(24) $$H_{(m)f}=\left\{ \begin{array}{l} 0,f<x(m-1)\\ \frac{f-x(m-1)}{x(m)-x(m-1)},x(m-1)\leq f\leq x(m)\\ \frac{x(m+1)-f}{x(m+1)-x(m)},x(m)<f\leq x(m+1)\\ 0,f>x(m+1) \end{array}\right.$$

where *m* is the filter bank number and the number of filters in this paper is set to 26; therefore, the range of *m* is 0<m<26 , the center frequency of the Mel filter. The formula for the calculation of x(m) is:

(25) $$\begin{array}{l} x(m)=(\frac{N}{f_{s}})Mel^{-1}(Mel(f_{\min})+\\ m\frac{Mel(f_{\max})-Mel(f_{\min})}{M+1} \end{array}$$

where $f_{s}$ is the sampling frequency, $f_{\max}$ , $f_{\min}$ represent the frequency range of the Mel filter bank, *N* is the number of S-transform samples, and M is the number of Mel filters.

The improved MFCC feature extraction method is distinguished from MFCC by the simpler operations of frame splitting and window adding. The specific steps are as follows:

(1) Framing: the S-transform has a high time complexity, so in order to save time, the original signal is framed with a fixed frame length.
(2) S-transform: the S-transform is performed on each frame by Equation (16) to obtain the time-frequency matrix A(t,f) .
(3) The spectral information is sought, as shown in Equation (26).
(26) $$F(f)=\frac{\sum_{i=1}^{t}{\left|A(t,f)\right|}^{2}}{t}$$
  where A(t,f) is the time-frequency matrix, *t* is the time corresponding to the S-transform matrix, and *f* is the frequency.
(4) Bandpass filtering is performed, as in Equation (27).
(27) $$Mel(m)=\ln(\sum_{k=0}^{N-1}\left|F(f)\right|H_{m}(f))$$
  where Mel(m) is the Mel filter output and $H_{m}(f)$ is the filter bank.
(5) A discrete cosine transform is performed as in Equation (28) to obtain the first set of voiceprint characterization coefficients $feat_{1}$ .
(28) $$C(i)=\sum_{j=1}^{m}Mel(m)\cos(\frac{\pi i(m-0.5)}{26})$$
(6) We perform first-order and second-order differentiation operations on $feat_{1}$ to obtain the second and third sets of parameters $feat_{2}$ , $feat_{3}$ of the improved MFCC eigenvectors.
(7) We splice the three sets of parameters to form the feature vector $\mathrm{IMFCC}=[feat_{1},feat_{2},feat_{3}]$ .

**Figure 7:**
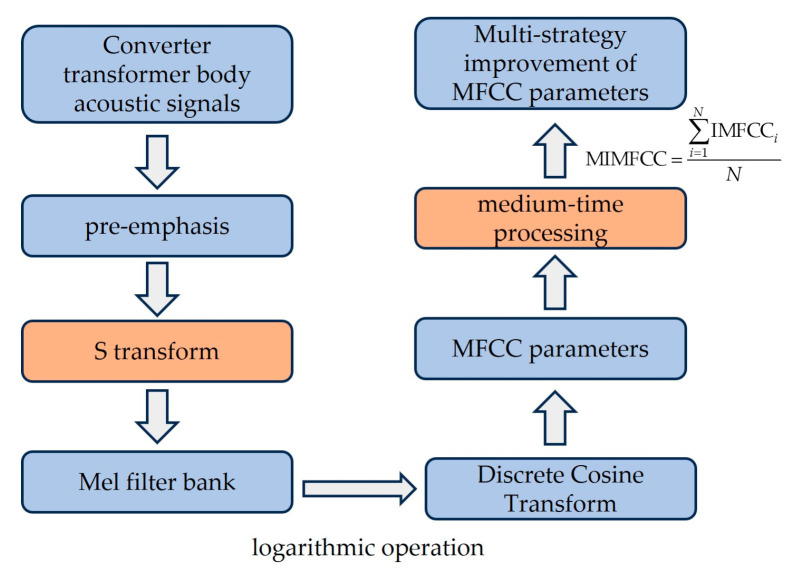
Multi-strategy improvement of MFCC flowchart.

Compared with the human speaking voice, power equipment voiceprint signal characteristics tend to be stationary; the feature vector obtained above contains a large amount of redundant information between the frames, so the use of mid-time features as shown in Equation (29) is more in line with the characteristics of stationary power equipment voiceprint features, reducing the interference of the heterogeneous long frames and having a stronger generalization [27], The multi-strategy improvement MFCC flowchart is shown in Figure 7.

(29) $$MIMFCC=\frac{\sum_{i=1}^{N}IMFCC_{i}}{N}$$

where $IMFCC_{i}$ is the *i*th frame signal feature and *N* is the number of medium-time signal frames and denotes MIMFCC is the medium-time feature vector.

### 3.4. Improved Temporal Convolutional Neural Networks

Time convolutional networks have good sequence information processing capabilities. In comparison to traditional architectures such as convolutional neural networks, this network achieves deeper networks by incorporating skip connections of residual blocks, effectively integrating shallow features into the depths for improved accuracy [28,29]. To simplify the network’s complexity, cavity convolution is employed to expand the sensory field, and the causal cavity convolution is calculated as shown in Equation (30):

(30) $$F(t)=\sum_{i=0}^{k-1}f(i)x_{t-di}$$

where *d* is the void coefficient, *k* is the convolution kernel size, and f(i) is the *i*th element of the convolution kernel.

The traditional TCN residual module introduces nonlinearity through the Relu activation function. However, when the input is negative, the zero-gradient problem occurs, leading to the offset phenomenon. This, in turn, limits the learning efficiency and effectiveness of the TCN. Setting the output mean of the activation function to zero serves a dual purpose: it reduces the gradient vanishing problem and mitigates the impact of weight initialization. Additionally, the output of the activation function with zero-mean facilitates the propagation of information between the different layers of the network, resulting in better learning dynamics. This helps the network learn complex features and representations more efficiently. To a greater extent, it can enhance the network’s learning performance. Therefore, the Mish activation function is used to replace the traditional Relu function, as in this equation:

(31) $$F(x)=\mathrm{mish}(x)=x\times \tanh(\ln(1+e^{x}))$$

**Figure 8:**
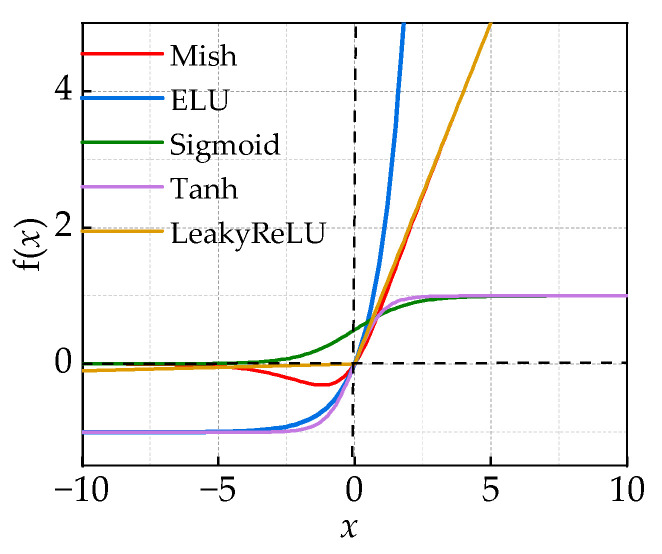
Activation function 0-mean comparison.

As depicted in Figure 8, compared with other activation functions, although the Tanh function has an absolute 0-mean value, it is prone to gradient vanishing due to the range of [−1, 1]. The Mish activation function is a better trade-off between the 0-mean value and the gradient vanishing problem [30].

**Figure 9:**
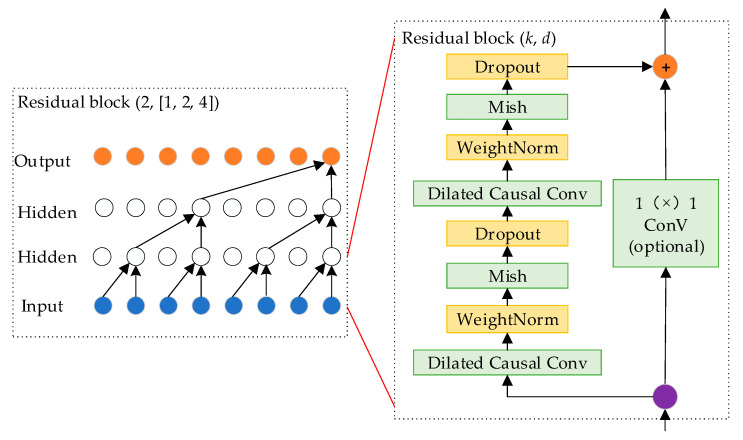
Improve temporal convolutional neural network architecture.

The improved TCN architecture is illustrated in Figure 9 (*k* = 2, *d* = 1, 2, 4), where each residual module contains two causal convolutional layers. The network’s performance is enhanced through the incorporation of the Mish activation function, weight normalization, and dropout.

The improved TCN pseudocode is shown in Algorithm 2:
**Algorithm 2** improved Temporal Convolutional Network
**Input:** Input sequence *X* with length *T*, Number of residual blocks *K*, Stack size *S*, Number of output channels *C*, Filter size *f*, Initial dilation value *d*_0_, Learning rate *η*

**Output:** Probability distribution over classes 
1: Initialize all model parameters 
2: Set learning rate to *η*

3: Set initial dilation value to *d*_0_

4: **for**
*k* = 1 **to** *K* **do**

5:   **for** *s* = 1 **to** *S* **do**
6:     **for** *c* = 1 **to** *C* **do**

7:       Apply causal convolution to input sequence *X* with dilation *d*

8:       Apply activation function (e.g., Mish) to the output 
9:       Apply weight normalization to the output 
10:      Update output sequence *O*

11:     **end for**

12:    **end for**

13:    Stack the output sequence *O* with the input sequence *X* as the new input 
14:    Increase the dilation value *d* exponentially 
15: **end for**

16: Apply a fully connected layer to the final output sequence *O*

17: Apply softmax function to obtain probability distribution over classes

### 3.5. Multi-Strategy Improved MFCC-IHPO-VMD-ITCN Combined Fault Diagnosis Modeling

**Figure 10:**
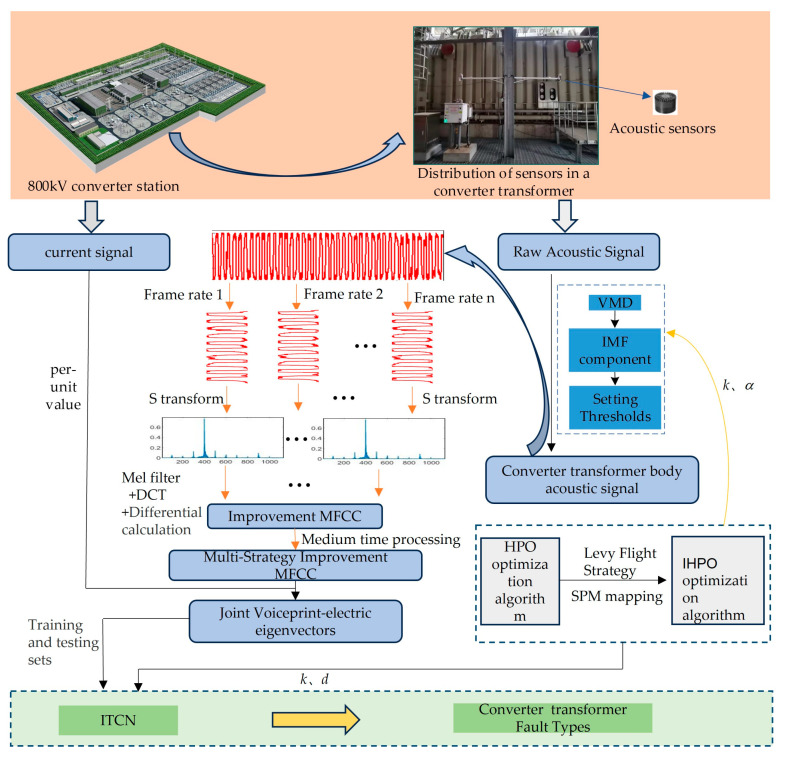
Multi-strategy improved MFCC-IHPO-VMD-ITCN fault diagnosis modeling.

Converter transformer voiceprint signals are mainly concentrated in the low-frequency band. Considering the operating patterns of the converter transformer, a combined voiceprint–electric feature vector is adopted to overcome the problem of interference between core and winding vibrations. The accurate identification of converter transformer faults is achieved through a diagnostic process from denoising through feature extraction to pattern recognition. The diagnostic workflow is illustrated in Figure 10.

The VMD is optimized based on IHPO to obtain the proprioceptive voiceprint signal. The selection of the decomposition number *k* and the penalty factor *α* has a significant impact on the decomposition result. It is prone to over-decomposition or loss of band information. Therefore, the minimum envelope entropy shown in Equation (32) is selected as the fitness function. IHPO is utilized to select the optimal [*k*, *α*] to overcome the inherent defects of VMD decomposition.

(32) $$\left\{ \begin{array}{l} Fitness=\min(f(i))\\ f(i)=-\sum_{i=1}^{N}p(i)\cdot \log10(p(i))\\ p(i)=a(i)/\sum_{i=-1}^{N}a(i) \end{array}\right.$$

where *N* is the number of Intrinsic Mode Function (IMF) components, f(i) is the envelope entropy after Hilbert adjustment, p(i) is the normalized form, and a(i) is the envelope signal.

Through the normalization of the load signal combined with the construction of multi-strategy improved MFCC for converter voiceprint and electric joint feature vector, multi-channel signal fault diagnosis is achieved.

Optimizing ITCN based on IHPO involves fine-tuning key parameters like kernel size (*k*) and dilation factor (*d*) for expansion convolution, which are crucial in determining the receptive field size and training accuracy. Utilizing Equation (33) as the fitness function enables adaptive optimization of ITCN to find optimal values for (*k*) and (*d*) that maximize the performance.

(33) Fitness=(1−accTrain)×100

where accTrain is the training set accuracy.

## 4. Calculus Analysis

### 4.1. Noise Reduction Processing for Voiceprint Signals

The voiceprint signals collected from outdoor substations are susceptible to significant transient and continuous noise interference, which inevitably affects the accuracy of fault diagnosis. Therefore, performing noise reduction processing is crucial.

**Figure 11:**
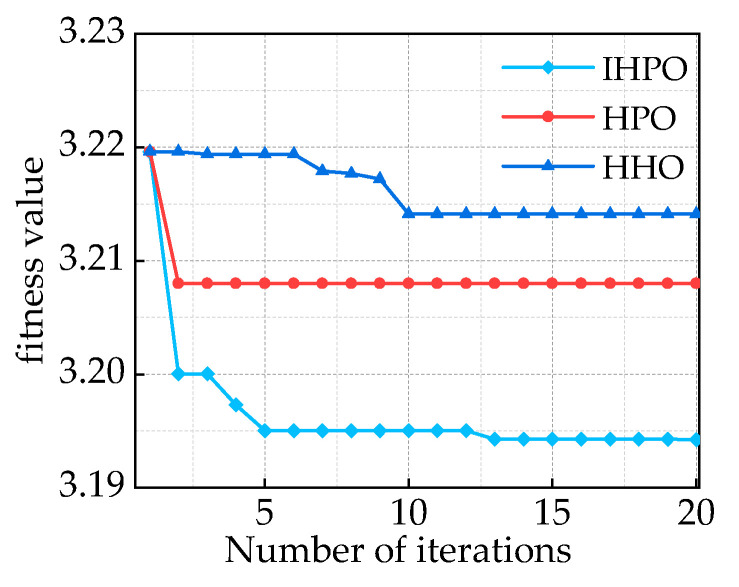
Comparison of fitness function values of different optimization algorithms.

Based on the given information, the optimization algorithm has a population size of 25 and a dimension of 2. The upper limit is denoted as $u_{a}=[25,3000]$ , while the lower limit is denoted as $u_{b}=[1,500]$ . Through 20 iterations, the fitness function changes are shown in Figure 11. In comparison to the HPO and HHO algorithms that converge to 3.208 and 3.2141, respectively, the proposed IHPO optimization algorithm in this study demonstrates better convergence performance.

**Figure 12:**
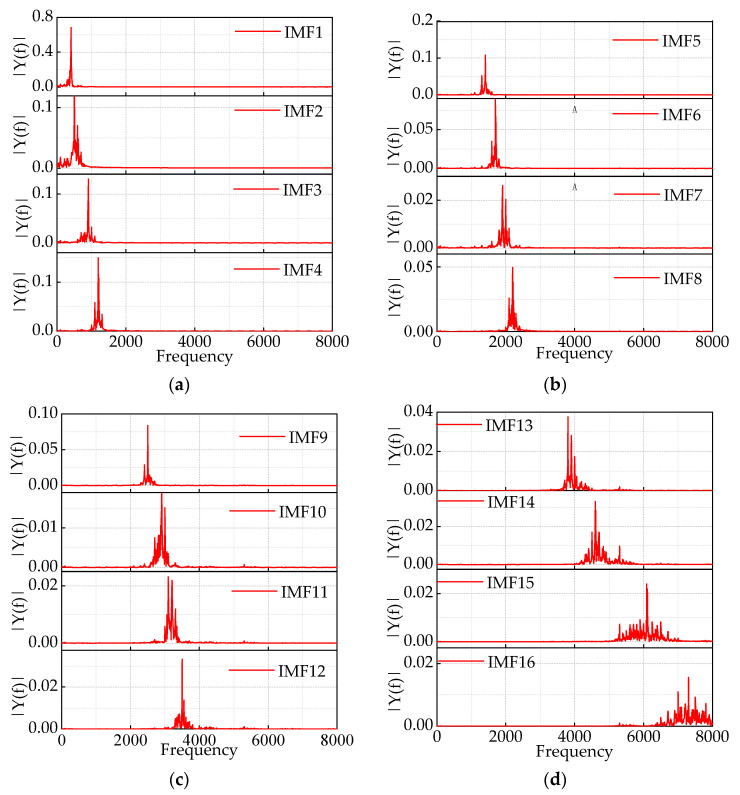
(**a**–**d**) Component IMF1–IMF16 after IHPO-VMD decomposition.

It reaches the optimal solution within five iterations, with a significantly smaller final fitness value of 3.194. The optimal values obtained are *k* = 16 and *α* = 1246. The results of the IMF decomposition using IHPO-VMD are shown in Figure 12.

**Figure 13:**
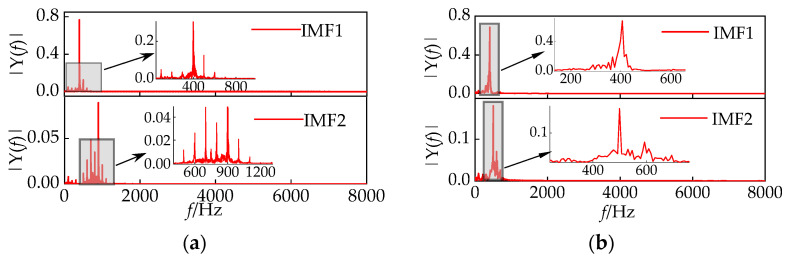
(**a**) Empirically selected VMD decomposition results; (**b**) IHPO-VMD decomposition results.

This article compares the results of IHPO-VMD with manually selected values of *k* and *α* to validate the superiority of IHPO-VMD. Taking *k* = 16 and *α* = 1000 as an example, the first two decomposition results are shown in Figure 13a. When the value of *α* is too small, it results in a wide bandwidth, causing severe mode mixing between the 400 Hz and 500 Hz components, as well as between the 600 Hz and 1000 Hz components. In contrast, Figure 13b shows that IHPO-VMD avoids the mode mixing problem.

**Figure 14:**
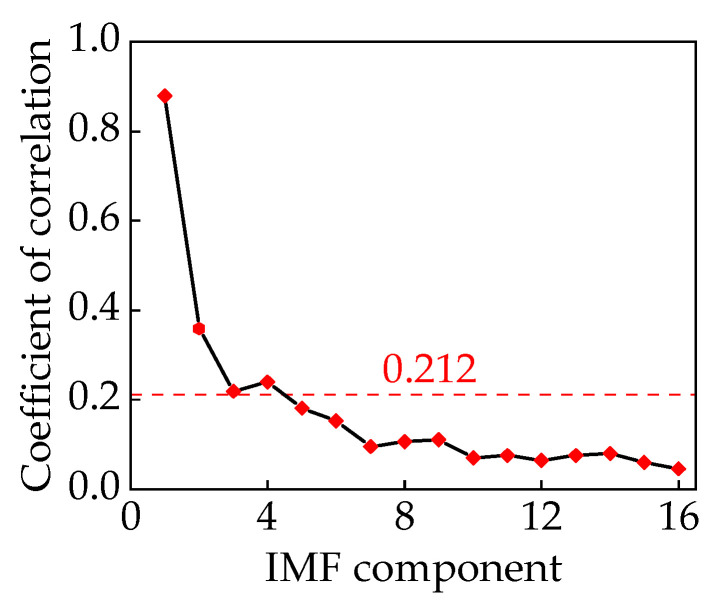
Correlation coefficients of components.

By calculating the correlation coefficients of the 16 IMF components, noise reduction processing can be achieved by setting a threshold using Equation (34). The correlation coefficients of each component are illustrated in Figure 14. Through the establishment of a threshold value, C=0.212 , the IMF1–IMF4 components can be recombined to derive the voiceprint signal of the converter transformer.

(34) $$\left\{ \begin{array}{l} C=\sqrt{\frac{\sum_{i=1}^{n}{\left(\rho_{i}-\bar{\rho }\right)}^{2}}{k}}\\ \rho_{k}=\frac{\sum_{i=1}^{n}\left(x_{i,k}-{\bar{x}}_{k}\right)\left(y_{i}-\bar{y}\right)}{\sqrt{\sum_{i=1}^{n}{\left(x_{i,k}-{\bar{x}}_{k}\right)}^{2}}\sqrt{\sum_{i=1}^{n}{\left(y_{i}-\bar{y}\right)}^{2}}} \end{array}\right.$$

where $\rho_{i}$ is the correlation coefficient of the *i*th order IMF component, $\bar{\rho }$ is the mean value, *k* is the number of components, x is the IMF component; y is the original signal; and *n* is the number of sampling points.

### 4.2. Joint Feature Vector Extraction

**Figure 15:**
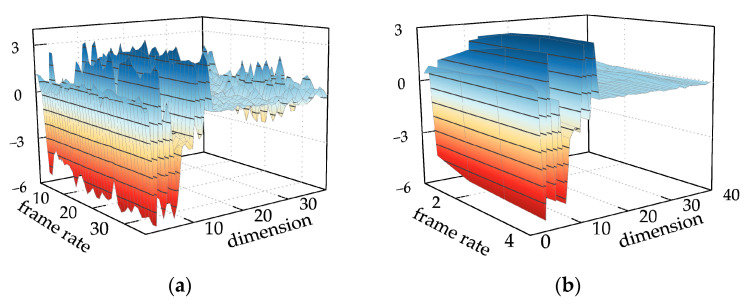
(**a**) Traditional MFCC features; (**b**) multi-strategy improved MFCC features.

Based on the 1 s denoised voiceprint data, a frame length of 25 ms was chosen to generate an enhanced MFCC feature vector with a size of [36×39] . In this representation, 36 denotes the number of frames, and 39 signifies the dimensionality of the feature vector, as depicted in Figure 15a. The voiceprint signal of the converter transformer demonstrates stability, exhibiting high redundancy between frame numbers. To mitigate complexity, a mid-term feature vector of 250 ms was constructed, as depicted in Figure 15b, where the feature vector changes from [36×39] to [4×39] . This leads to a notable reduction in its complexity.

**Figure 16:**
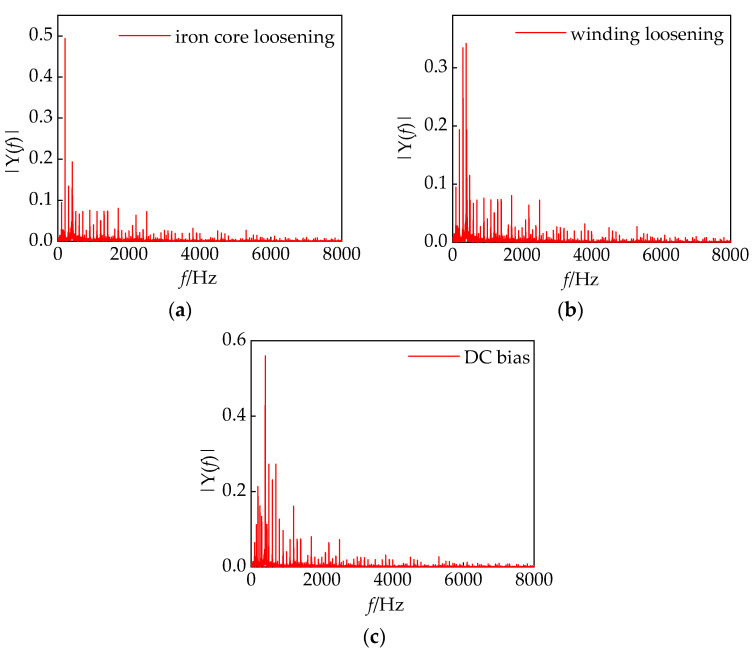
(**a**–**c**) are the typical defective spectral characteristics of the converter transformer.

Based on the provided information, feature extraction from the load signal was carried out using per-unit value to construct a joint voiceprint–electric feature vector of size [4×40] . The iron core faults and winding faults in the converter transformer exhibit strong randomness, with distinct characteristic spectra corresponding to different loosening conditions. As analyzed in 2.3 and Table 1, iron core fault diagnosis is accomplished in Stage I, while winding fault diagnosis is achieved in Stage III. Through the separation of voiceprint features of the iron core and winding based on the load signal, a joint voiceprint–electric feature vector is constructed. In Stage II, the fault is defined as either an iron core or winding fault. However, this stage represents an unmonitorable phase, and determining whether a core failure or a winding failure is challenging for maintenance personnel. The fault diagnosis is conducted in stages to precisely identify iron core loosening faults, winding loosening faults, and DC bias faults. This approach effectively overcomes the limitation of existing research focusing on single fault diagnosis, providing a more comprehensive diagnostic capability. The spectral characteristics of typical defects in the converter transformer section are illustrated in Figure 16.

### 4.3. Description of Experimental Objects and Measurement Points

The converter transformer, operating at a high voltage level and featuring a complex structure, plays a crucial role in high-voltage DC transmission technology. Utilizing the original model for fault diagnosis studies involves significant expenses and requires extensive equipment. Therefore, in this study, we sourced fault data from the signal detection system of an 800 kV converter station mentioned above. This system not only enables real-time storage of fault data but also allows for historical playback. To diversify fault samples, we used the monitoring system to collect fault signals from other converter stations to build a sample library. In this article, we collected fault signals from converter transformers experiencing DC bias, core loosening, winding loosening, and normal states. Both acoustic and current signals were collected through historical playback. The dataset was constructed following the method outlined in Section 4.2, involving division into the training sets and the test sets to ensure the effectiveness of deep learning [31], as shown in Table 3.

**Figure 17:**
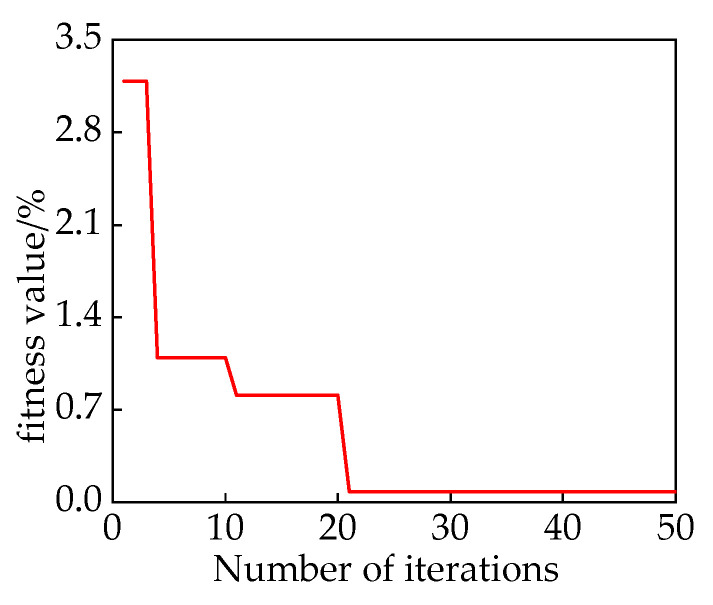
Change in fitness function.

The IHPO parameters were set as follows: the number of populations is 30, the maximum number of iterations is 50, the epoch of parameter optimization is 50, the upper limit is ua=[16,6] , and the lower limit is ub=[1,1] . Adaptive optimization of the convolution kernel size *k* and the expansion factor *d* was realized, and the change in fitness value is shown in Figure 17, which converged to 0.082 after 21 iterations and gave outputs of *k* = 16 and *d* = 3.

**Figure 18:**
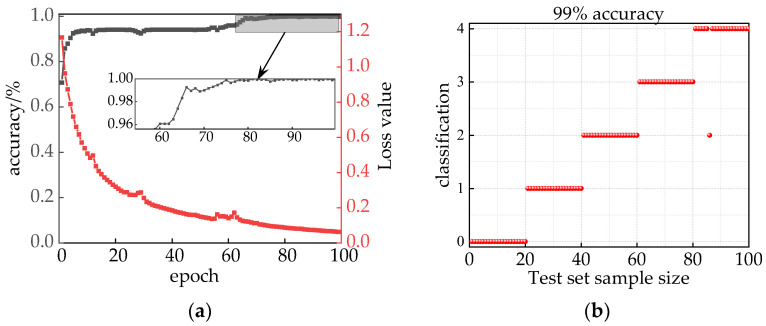
(**a**) Model identification results; (**b**) model testing set prediction results.

The optimized results of IHPO were used as the input for ITCN, configuring the model with an epoch set to 100 and a batch size of 32. As illustrated in Figure 18a, this integrated model demonstrated stable convergence, achieving 100% accuracy after 88 epochs. To validate the training accuracy of the model, it was tested using a validation set, and the prediction results are depicted in Figure 18b, with a test accuracy of 99%. Through this analysis, the combination model, which utilizes current signals and incorporates audio–electric joint features, successfully mitigated interference between faults, affirming the feasibility of this combined model.

### 4.4. Comparative Analysis of Combined Forecasting Methods

**Figure 19:**
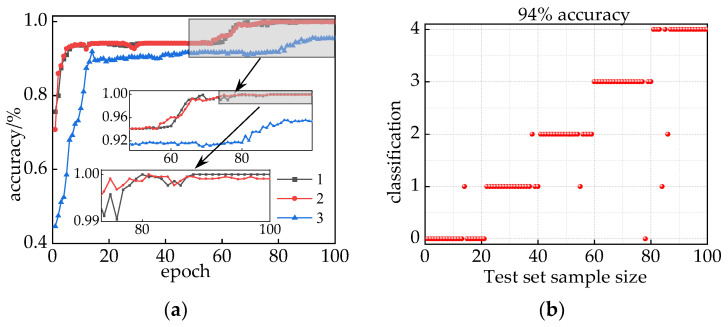
(**a**) 1 IHPO-ITCN based on joint voiceprint–electric feature vectors, (**a**) 2 IHPO-TCN based on joint voiceprint–electric feature vectors, (**a**) 3 IHPO-ITCN based on voiceprint feature vectors; (**b**) prediction results of the voiceprint feature model testing set.

To assess the performance of the combined model, in this article, we conducted a comparison with the IHPO-TCN model utilizing voiceprint–electric joint feature vectors and the IHPO-ITCN model based on audio feature vectors. The results are presented in Figure 19a. Upon comparing a (1) and a (2), it is evident that a (1) exhibits superior convergence, reaching 99.91% accuracy as epoch increases, surpassing a (2) in stability. This validates the superiority of model (a). In contrast, model a (3) achieves lower accuracy, converging to 95.41% after 94 epochs. The test set prediction results for the a (3) model are depicted in Figure 19b, with a test accuracy of 94%. Notably, mixed interference between core loosening and winding loosening faults is observed. In conclusion, the IHPO-ITCN model based on audio-electric joint feature vectors demonstrates significant superiority compared to the other models analyzed.

A comparison of training time and accuracy of different feature signal fault recognition models is shown in Table 4. In the comparison experiments, the number of training sets and test sets are shown in Table 3, and the parameter settings of each model are also equal. Compared with traditional MFCC, MFCC’s multi-dimensional improvement strategy decreased training time by 26 s and increased accuracy by 2.82%. These results validate the superiority of the improved MFCC in feature extraction. Due to changes in feature dimensions, the training time of feature vectors constructed by the voiceprint signals’ combined load is longer. Compared with traditional MFCC features, traditional MFCC combined load features have a longer training time of 5.6 s but an accuracy improvement of 5.95%. Similarly, multi-strategy improvement MFCC combined load features have a training time increase of 1.1 s but an accuracy improvement of 4.33% compared to single multi-strategy improvement MFCC features. This verifies that although load signal intervention prolongs a certain training time, it effectively improves the accuracy of fault classification. For the diagnostic model proposed in this article, the accuracy ultimately converges to 100% and the training time is shorter, thus confirming the superior performance of the model.

In order to further substantiate the superiority of TCN in inverter voiceprint fault diagnosis, in this article, we conducted a comparative analysis with traditional machine learning algorithms, ensuring consistency in dataset determination, epochs, and other parameters used for the comparison method. The hyperparameter settings of the comparison model are provided in Table 5.

The recognition results of different machine learning models are presented in Table 6: Utilizing the load joint multi-strategy to improve MFCC parameters as fault features to construct a dataset, the four machine learning algorithms show good results in training time and test set recognition accuracy, further verifying the effectiveness of the fault diagnosis model in feature extraction. However, when compared with CNN, although TCN has a training time of 1.9 s longer, it excels in capturing deep features, leading to a 3% higher recognition accuracy. In contrast to TCN, the training times of GRU and LSTM are 2.2 s and 2.7 s longer, respectively, with accuracy reductions of 7% and 5%, confirming the superiority of TCN in this diagnostic model.

## 5. Conclusions

This paper proposes a fault diagnosis method that combines the multidimensional-improvement strategy of MFCC with adaptive VMD-ITCN and incorporates the influence of load signals. This method significantly enhances recognition accuracy and is applicable in the field of fault diagnosis for converter transformers. Our experimental results demonstrate that the application of IHPO for optimizing VMD and ITCN has significant benefits, such as improved convergence and the avoidance of parameter-related impacts on fault diagnosis models. The introduction of load signals divides the entire operational process of the converter transformer into three stages, diagnosing core faults in Stage I and winding faults in Stage III. The effectiveness of the proposed model was verified using a sample dataset from an 800 kV converter station. This model exhibits superior performance in terms of recognition accuracy and training speed, providing a new approach for maintenance personnel to promptly and accurately detect internal defects in converter transformers.

The fault diagnosis model proposed in this article is based on a data-driven background, which achieves fault classification through row analysis of historical data of converter transformers. Therefore, the number of fault categories and samples is relatively small. In future research, we will collect fault data of converter transformers in different scenarios and expand the types of faults. The idea of transfer learning, as described in reference [32,33], can also be introduced to further improve the generalization of diagnostic models. On the other hand, we will consider establishing an accurate mathematical model from a model-driven perspective to simulate fault signals and achieve fault diagnosis.

## Figures and Tables

**Table 1:** Parameters of converter transformer.

Parameters	Numerical Value	
	Pole II High-End Y/D Converter	Pole I High-End Y/Y Converter
Model number	ZZDFPZ-412300/600 kV	ZZDFPZ-412300/750/800
Rated capacity/MVA	412.3	412.3
Net side $I_{N}/A$	933	933
Valve side $I_{N}/A$	2357	4083
Operating frequency/Hz	50	50
Cooling method	OFAF	OFAF

**Table 2:** Acousto-electric signal correlation analysis.

Operational State	No-Load (I)	Load (II)	Load (III)
Current and voltage signals	U = 1 I = 0	U = 1 I < 0.23	U = 1 I > 0.23
Voiceprint signal main frequency/Hz	200	200/400	400
Conclusion	Iron core vibration dominated	The core windings alternately dominate	Winding vibration dominant

**Table 3:** Combined model training program.

Operational State	Serial Number	Training Sets/Each	Test Sets/Each
Normal	0	180	20
Iron core loosening	1	180	20
Winding loosening	2	180	20
DC bias	3	180	20
Core or winding fault	4	180	20

**Table 4:** Comparison of training time and accuracy of different feature signal fault recognition models.

Characteristic Signal Type	Training Time/s	Convergence to Maximum Accuracy/%
Traditional MFCC	50.6	92.85
Multi-strategy improvement MFCC	24.6	95.67
Load + multi-strategy improvement MFCC	25.7	100
Load + traditional MFCC	56.3	98.8

**Table 5:** Contrasting model hyperparameter settings.

Contrast Model	Activation Function	Batch Size	Learning Rate
TCN	Relu	16	0.001
CNN	Relu	16	0.001
LSTM	Relu	16	0.001
GRU	Relu	16	0.001

**Table 6:** Comparison model recognition results.

Contrast Model	Training Time/S	Test Set Accuracy/%
TCN	25.7	99
CNN	23.8	96
LSTM	27.9	92
GRU	28.4	94

## Data Availability

The data are contained within the article.
